# Pipeline for Annual Averaged Wind Power Output Generation Prediction of Wind Turbines Based on Large Wind Speed Data Sets and Power Curve Data

**DOI:** 10.1016/j.mex.2021.101499

**Published:** 2021-08-25

**Authors:** Benjamin Wacker, Jan Chr. Schlüter

**Affiliations:** a Department of Engineering and Natural Sciences, University of Applied Sciences Merseburg, Eberhard-Leibnitz-Str. 2, Merseburg D-06217, Germany; b Next Generation Mobility Group, Max-Planck-Institute for Dynamics and Self-Organization, Department of Dynamics of Complex Fluids, Am Fassberg 17, Göttingen D-37077, Germany; c Institute for Dynamics of Complex Systems, Faculty of Physics, Georg-August-University of Göttingen, Friedrich-Hund-Platz 1, Göttingen D-37077, Germany; d Flexible Transport Systems and Complex Urban Dynamics Research Group, “Friedrich List” Faculty of Transport and Traffic Sciences, Technical University of Dresden, Hettnerstr. 1, 01069 Dresden, Germany; e Econophysics Lab, Chair for Network Dynamics, Center for Advancing Electronics Dresden (cfaed), Technical University of Dresden, Helmholtzstr. 18, 01069 Dresden, Germany

**Keywords:** Data Analysis, Energy Analysis, Power Curve Modeling, Power Output Generation, Wind Energy, Wind Speed Probability Distributions

## Abstract

In this article, an abstract framework for annual averaged wind power output generation prediction of wind turbines is presented which is heavily based on large wind speed data sets and power curve data of wind turbines due to the rising interest in wind energy as one main future renewable energy source. As combinations of arbitrary power curve modeling techniques and arbitrary wind speed distributions based on wind speed data are seldom combined, the abstract combination of these two aspects in wind power output generation prediction in one pipeline is thoroughly described here. Conclusively, one detailed example wind speed data set from a weather station situation in Bremen, Germany illustrates applicability of the presented framework.

## Graphical abstract

**Figure:**
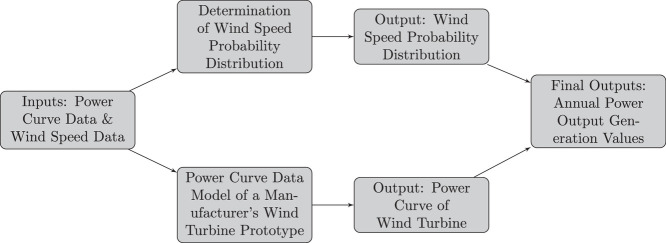

Specifications table

**Table:** 

*Subject area:*	Energy
*More specific subject area:*	Wind Energy
*Method name:*	Wind Power Output Generation Prediction
	Algorithm for Wind Turbines
*Name and reference*	Jung, C. (2016). High Spatial Resolution Simu-
*of original sources:*	lation of Annual Wind Energy Yield Using Near-
	Surface Wind Speed Time Series. *Energies*,
	9(5): 344, DOI: 10.3390/en9050344. [1]
	(As a review for wind speed
	probability distributions)
	Pei, S., Li, Y. (2019). Wind Turbine Power Curve
	Modeling with a Hybrid Machine Learning
	Technique. *Applied Sciences*, 9: 4930,
	10.3390/app9224930. [2] (As a review for
	power curve modeling techniques)
	Wacker, B., Seebaß, J., Schlüter, J. (2020).
	A Modular Framework for Annual Averaged
	Power Output Generation of Wind Turbines. *Energy*
	*Conversion and Management*, 221:113149,
	DOI: 10.1016/j.enconman.2020.113149. [3]
	(As the main source for the
	original method)
*Resource availability:*	There are no special resources.

## Introduction

Interest in wind energy as one main future renewable energy source has risen constantly over the past years [3]. Due to this rising importance of wind energy as one major ingredient to reduce carbon dioxide emissions, prediction methods for wind power output are valuable tools for determination of wind turbine locations. For this purpose, one must model power curves from given power curve data from manufacturers as summarized in [2]. Additionally, one needs to further identify possible wind speed distributions from given large wind speed data sets [1], [4]. Based on these articles, Wacker, Seebaß and Schlüter proposed an abstract framework for annual averaged wind power output generation prediction of wind turbines which heavily relies on large wind speed data sets and power curve data of wind turbines [3] because these methods are often considered separately in the literature, and can scarce be found combined. However, it seems important to present algorithmic aspects of the aforementioned article in greater details.

For these reasons, we present a complete pipeline for annual averaged wind power output generation prediction of wind turbines in this article which was first developed in [3]. This method relies heavily on large wind speed data sets, arbitrary power curve modeling techniques and arbitrary wind speed distributions. Finally, we provide one detailed example from a weather station situation in Bremen, Germany.

As already mentioned, prediction of produced energy by a wind turbine is an important topic because renewable energy sources are necessary to reduce carbon dioxide emissions. In this work, we provide details regarding our abstract pipeline’s framework for this goal. The following steps are necessary ingredients.

• **Step 1:** Since wind speed data sets may come from different sources, different pre-processing steps need to be taken into account. This includes adjusting wind speeds at different heights by so-called power laws.
• **Step 2:** Different power curve models might be adapted to given power curve data.
• **Step 3:** We choose different wind speed probability distributions to fit our processed wind speed data.
• **Step 4:** As our main output, we approximate integrals by finite sums to calculate semi-empirical and estimated wind power output generation prediction values numerically.
• **Step 5:** We suggest different goodness-of-fit measures for evaluation purpose.

## Method details

**Fig. 1:**
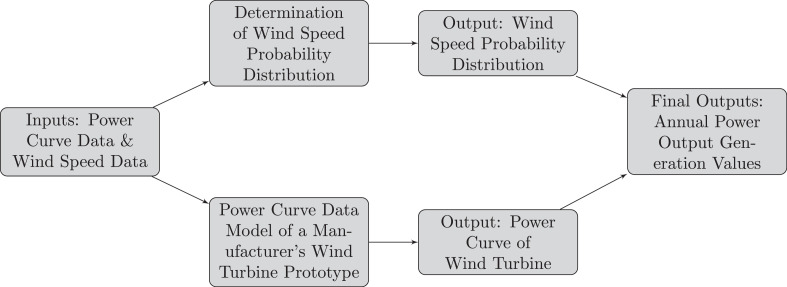
A flowchart of our abstract framework for annual averaged power output generation prediction

Let ${\left\{v_{j}\right\}}_{j=1}^{N}$ be a time series of measured wind speed at a certain weather station. Let $\left(v_{k},P_{k}\right)$ be measured power curve data of a manufacturer’s wind turbine prototype. These data sets build our foundation for our wind power output generation prediction algorithm. We portray the graphical flowchart of our algorithm in Figure 1. All steps coincide with our procedure presented in our abstract. We mainly follow our preprint but add further details regarding our methods. However, we especially discuss power curve modeling and uncertainty quantification in a more detailed manner.

### Step 1: Processing of wind speed data sets

In our complete pipeline, we process wind speed data sets provided by the German Weather Service (DWD) [5] and National Centers for Environmental Information [6]. Since both data sets differ, we have to adjust our processing steps accordingly.

Let us first consider wind speed data sets from the German Weather Service. We take a closer look at data from the weather station located at Bremen, Germany (Station ID: 00691). Data can be extracted from the corresponding ZIP-archive and data are contained in the text-file named produkt_ff_stunde_00691.txt. The fourth column consists of measured wind speed with physical unit $\frac{\text{m}}{\text{s}}$.



Missing data are replaced by −999. This fact implies that we have to delete these entries from our code. As an outlook, we also need to delete zero wind speed values from this column for estimation of two-parameter Weibull distributions (compare Step 3).

International data sets from National Centers for Environmental Information need different treatment. A short extract of such files is given below.



The ninth column contains wind speed values scaled by a factor of ten. For this reason, we have to rescale these data by dividing these values by a factor of ten. Since international data sets are archived for every year, we must put together complete time series. If we want to adjust the given wind speeds of the weather stations at reading height to hub height, we need so-called power laws [7], [8]. If $h_{r}$ is the reading height and $v_{r}$ is the measured wind speed at reading height, the extrapolation power law for the new wind speed v at hub height h reads

$$v=v_{r}\cdot {\left(\frac{h}{h_{r}}\right)}^{\alpha }$$

where α is an empirical coefficient depending on the location’s roughness. For further details, we refer interested readers to [7], [8]. Concluding this step, we provide short pseudo-code which describe our wind speed processing procedure in Algorithm 1.

**Algorithm 1:**
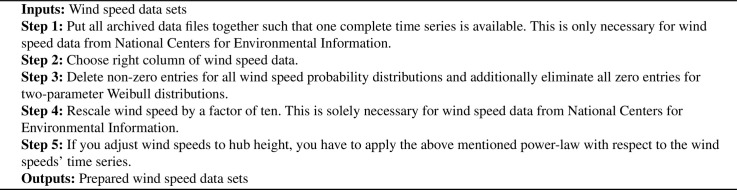
Pseudo-code for wind speed data processing

Since all following steps are the same for different time series of wind speeds, we restrict our discussion and results to the case of wind speeds measured at reading height. However, all our mentioned steps can still be carried out if we apply the power law to the wind speeds at our pre-processing step 1.

### Step 2: Power curve modeling

**Fig. 2:**
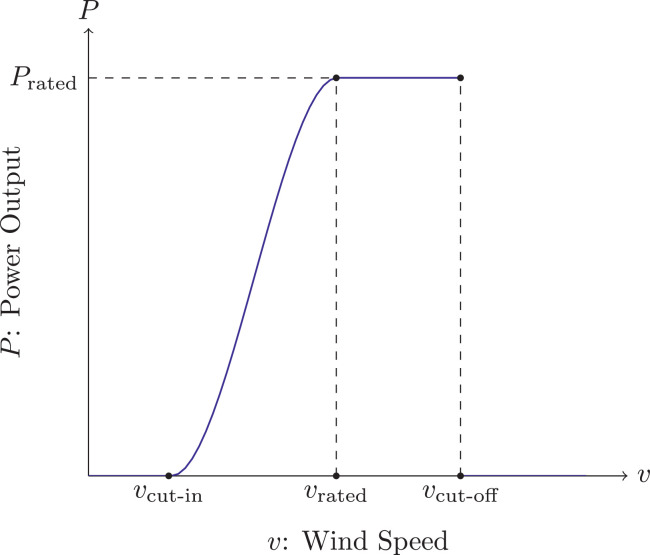
Plot of a general wind power curve

A typical course of wind power curves is shown in Figure 2.

We observe that wind power curves can be described by piecewise defined functions. This general approach reads

(1) $$P_{\text{Power}}\left(v\right)=\left\{ \begin{array}{cc} 0 & ,v\in \left[0,v_{\text{cut-in}}\right)\\ q\left(v\right) & ,v\in \left[v_{\text{cut-in}},v_{\text{rated}}\right]\\ P_{\text{rated}} & ,v\in \left(v_{\text{rated}},v_{\text{cut-off}}\right)\\ 0 & ,v\in \left[v_{\text{cut-off}},\infty \right) \end{array}\right.$$

where q(v) is an arbitrary function on $\left[v_{\text{cut-in}},v_{\text{rated}}\right]$. Here, v represents wind speed while $v_{\text{cut-in}}$, $v_{\text{rated}}$ and $v_{\text{cut-off}}$ denote cut-in wind speed, rated wind speed and cut-off wind speed respectively. $P_{\text{rated}}$ is the rated power output.

Before considering power curve modeling in more detail, we summarize given wind speed data from manufacturer Vestas [9] in Table 1.

**Table 1:** Hourly Power Output Data For Wind Turbine Vestas V112

Wind Speed	Power Output	Wind Speed	Power Output
0.0	0	13.5	3075
0.5	0	14.0	3075
1.0	0	14.5	3075
1.5	0	15.0	3075
2.0	0	15.5	3075
2.5	0	16.0	3075
3.0	26	16.5	3075
3.5	73	17.0	3075
4.0	133	17.5	3075
4.5	207	18.0	3075
5.0	302	18.5	3075
5.5	416	19.0	3075
6.0	554	19.5	3075
6.5	717	20.0	3075
7.0	907	20.5	3075
7.5	1126	21.0	3075
8.0	1375	21.5	3075
8.5	1652	22.0	3075
9.0	1985	22.5	3075
9.5	2282	23.0	3075
10.0	2585	23.5	3075
10.5	2821	24.0	3075
11.0	2997	24.5	3075
11.5	3050	25.0	3075
12.0	3067	25.5	0
12.5	3074	26.0	0
13.0	3075	26.5	0

We clearly see that we can algorithmically determine $v_{\text{cut-in}}$, $v_{\text{rated}}$ and $v_{\text{cut-off}}$ from these data. Determination of $v_{\text{cut-in}}$ is portrayed in Algorithm 2.

**Algorithm 2:**
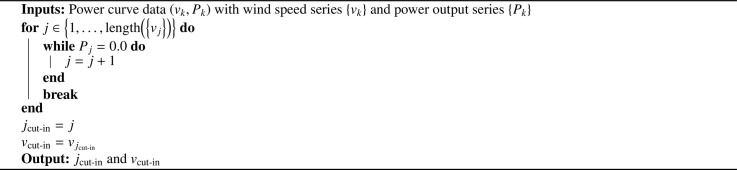
Pseudo-code for determination of $v_{\text{cut-in}}$

Determination of $v_{\text{rated}}$ is shown in Algorithm 3.

**Algorithm 3:**
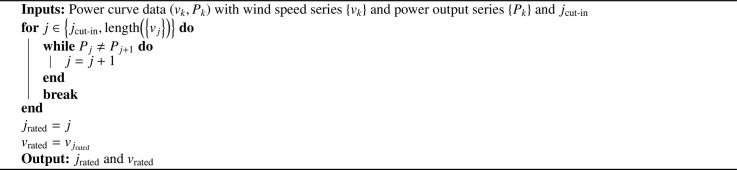
Pseudo-code for determination of $v_{\text{rated}}$

Finally, our procedure for calculation of $v_{\text{cut-off}}$ is given in Algorithm 4.

**Algorithm 4:**
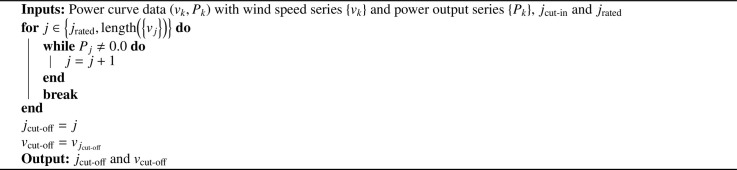
Pseudo-code for determination of $v_{\text{cut-off}}$

Now, we can use these results to interpolate power curve data points by certain power curve models. We restrict ourselves to two methods of cubic spline interpolation and logistic regression. For further models, we refer interested readers to [2].

At first, we begin with cubic spline interpolation. Let $v_{1},\ldots ,v_{M}\in \left[v_{\text{cut-in}},v_{\text{rated}}\right]$ be M ascending data points in the interval of interest, i.e.

$$v_{\text{cut-in}}=v_{1}<v_{2}<\ldots <v_{M-1}<v_{M}=v_{\text{rated}}.$$

Our cubic spline interpolation model $q_{\text{cub}}$ reads

(2) $$q_{\text{cub}}\left(v|\theta_{\text{cub}}\right)=\left\{ \begin{array}{cc} a_{1}\cdot v^{3}+b_{1}\cdot v^{2}+c_{1}\cdot v+d_{1} & ,v\in \left[v_{1},v_{2}\right)\\ a_{2}\cdot v^{3}+b_{2}\cdot v^{2}+c_{2}\cdot v+d_{2} & ,v\in \left[v_{2},v_{3}\right)\\ ... & ...\\ a_{M}\cdot v^{3}+b_{M}\cdot v^{2}+c_{M}\cdot v+d_{M} & ,v\in \left[v_{M-1},v_{M}\right] \end{array}\right.$$

where $a_{l},b_{l},c_{l},d_{l}$ for l∈{1,…,M} are all cubic interpolation parameters and $\theta_{\text{cub}}$ all summarizes them in one vector. To build the linear system, all data points have to be passed and first derivatives must be continuous. We further need to define appropriate boundary conditions. For further details on cubic spline interpolation methods, we refer interested readers to Fritsch and Carlson [10] or Hyman [11]. With respect to the scripting language R, all these variants are implemented by splinefun.

Let us now consider logistic regression. The logistic regression model function $q_{\text{log}}$ is defined by

(3) $$q_{\text{log}}\left(v|\theta_{\text{log}}\right)=\frac{B}{C+D\cdot \text{exp}\left(-E\cdot v+F\right)}$$

where B,C,D,E,F are all logistic regression parameters which are summarized in $\theta_{\text{log}}$. To apply ordinary least-squares regression, we define an optimization cost function J by

(4) $$J\left(\theta_{\text{log}}\right)=\sum_{j=1}^{M}{\left(q_{\text{log}}\left(v_{j}|\theta_{\text{log}}\right)-P_{j}\right)}^{2}$$

where $\left(v_{j},P_{j}\right)$ are given power curve data points. We refer interested readers to the optimization book of Nocedal and Wright for details on different algorithms to solve this problem formulation [12].

### Step 3: Wind speed probability distribution modeling

A given time series ${\left\{v_{k}\right\}}_{j=1}^{N}$ of N wind speed data points is our input for wind speed probability distribution modeling. Since we only can provide a non-exhaustive overview on this vast field, we refer interested readers to the review by Wang and co-authors [13].

We concentrate on three probability speed distribution models which are often applied in wind speed modeling [4] - two-parameter Weibull distributions, four-parameter Kappa distributions and five-parameter Wakeby distributions.

Let us start with the two-parameter Weibull distribution, the most-common used wind speed probability distribution in wind speed analytics. It is often obtained by maximizing log-likelihood functions. This method has favorable statistical properties. Recently, Wacker, Kneib and Schlüter also proved that this functional has a unique global maximizer [14]. Hence, numerical optimization simplifies in this case.

The two-parameter Weibull distribution reads

(5) $$p_{\text{Wei}}\left(v|A_{\text{Wei}},k_{\text{Wei}}\right)=\frac{k_{\text{Wei}}}{A_{\text{Wei}}}\cdot {\left(\frac{v}{A_{\text{Wei}}}\right)}^{k_{\text{Wei}}-1}\cdot \exp\left(-{\left(\frac{v}{A_{\text{Wei}}}\right)}^{k_{\text{Wei}}}\right)$$

for all v>0 where $A_{\text{Wei}}$ denotes the scale parameter and $k_{\text{Wei}}$ the shape parameter of the corresponding distribution [15]. The corresponding maximum log-likelihood function is defined by

(6) $$\begin{array}{cc} L\left(A_{\text{Wei}},k_{\text{Wei}}\right) & =N\cdot \text{ln}\left(k_{\text{Wei}}\right)-N\cdot k_{\text{Wei}}\cdot \text{ln}\left(A_{\text{Wei}}\right)+\left(k_{\text{Wei}}-1\right)\cdot \sum_{j=1}^{N}\text{ln}\left(v_{j}\right)\\ & \;\;\;\;-\sum_{j=1}^{N}{\left(\frac{v_{j}}{A_{\text{Wei}}}\right)}^{k_{\text{Wei}}}. \end{array}$$

We determine first derivatives of L by

(7) $$\frac{\partial L\left(A_{\text{Wei}},k_{\text{Wei}}\right)}{\partial A_{\text{Wei}}}=-\frac{N\cdot k_{\text{Wei}}}{A_{\text{Wei}}}+\sum_{j=1}^{N}\frac{k_{\text{Wei}}}{A_{\text{Wei}}}\cdot {\left(\frac{x_{j}}{A_{\text{Wei}}}\right)}^{k_{\text{Wei}}}$$

and

(8) $$\begin{array}{cc} \frac{\partial L\left(A_{\text{Wei}},k_{\text{Wei}}\right)}{\partial k_{\text{Wei}}} & =\frac{N}{k_{\text{Wei}}}-N\cdot \text{ln}\left(A_{\text{Wei}}\right)+\sum_{j=1}^{N}\text{ln}\left(v_{j}\right)\\ & \;\;\;\;-\sum_{j=1}^{N}\text{ln}\left(\frac{v_{j}}{A_{\text{Wei}}}\right)\cdot {\left(\frac{v_{j}}{A_{\text{Wei}}}\right)}^{k_{\text{Wei}}} \end{array}$$

respectively. If we set these equations equal to zero, we will obtain a nonlinear system of equations which can be solved, for example, by Newton methods [12]. We use such methods which are supplied by R-packages EnvStats [16] or **fitdistrplus** [17].

Let $\theta_{\text{Kap}}=\left(A_{\text{Kap}},k_{\text{Kap}},\mu_{\text{Kap}},h_{\text{Kap}}\right)$ be the summarizing vector of all four parameters for the Kappa distribution. The four-parameter Kappa distribution is then defined by

(9) $$p_{\text{Kap}}\left(v|\theta_{\text{Kap}}\right)=\frac{1}{A_{\text{Kap}}}\cdot {\left\{1-\frac{k_{\text{Kap}}\cdot \left(v-\mu_{\text{Kap}}\right)}{A_{\text{Kap}}}\right\}}^{\frac{1}{k_{\text{Kap}}-1}}\cdot {\left\{F_{{}_{\text{Kap}}}\left(v\right)\right\}}^{1-h_{\text{Kap}}}$$

for all v≥0 with scale parameter $A_{\text{Kap}}$, shape parameter $k_{\text{Kap}}$, location parameter $\mu_{\text{Kap}}$ and second shape parameter $h_{\text{Kap}}$. Here, the cumulative distribution function is given by

(10) $$F_{\text{Kap}}\left(v|\theta_{\text{Kap}}\right)={\left\{1-h_{\text{Kap}}\cdot {\left\{1-\frac{k_{\text{Kap}}\cdot \left(v-\mu_{\text{Kap}}\right)}{A_{\text{Kap}}}\right\}}^{\frac{1}{k_{\text{Kap}}}}\right\}}^{\frac{1}{h_{\text{Kap}}}}.$$

Finally, let $\theta_{\text{Wak}}=\left(A_{\text{Wak}},\gamma_{\text{Wak}},k_{\text{Wak}},\mu_{\text{Wak}},h_{\text{Wak}}\right)$ be the summarizing vector of all five parameters for the Wakeby distribution. The five-parameter Wakeby distribution is then defined by

(11) $$\begin{array}{cc} p_{\text{Wak}}\left(v|\theta_{\text{Wak}}\right) & =\{A_{\text{Wak}}\cdot {\left\{1-F_{\text{Wak}}\left(v\right)\right\}}^{\gamma_{\text{Wak}}-1}\\ & \;\;{+k_{\text{Wak}}\cdot {\left\{1-F_{\text{Wak}}\left(v\right)\right\}}^{-k_{\text{Wak}}-1}\}}^{-1} \end{array}$$

for all v≥0 with scale parameter $A_{\text{Wak}}$, second scale parameter $\gamma_{\text{Wak}}$, shape parameter $k_{\text{Wak}}$, location parameter $\mu_{\text{Wak}}$ and second shape parameter $h_{\text{Wak}}$. Here, the cumulative distribution function is implicitly given by

(12) $$\begin{array}{cc} F_{\text{Wak}}^{-1}\left(v|\theta_{\text{Wak}}\right) & =\mu_{\text{Wak}}+\frac{A_{\text{Wak}}}{\mu_{\text{Wak}}}\cdot \left\{1-{\left(1-F_{\text{Wak}}\left(v\right)\right)}^{\gamma_{\text{Wak}}}\right\}\\ & \;\;-\frac{k_{\text{Wak}}}{h_{\text{Wak}}}\cdot \left\{1-{\left(1-F_{\text{Wak}}\left(v\right)\right)}^{-h_{\text{Wak}}}\right\}. \end{array}$$

In contrast to two-parameter Weibull and four-parameter Kappa distributions, this implies that five-parameter Wakeby distributions are only implicitly defined. An often applied method to estimate parameters in four-parameter Kappa and five-parameter Wakeby distributions is the estimation method of L-moments. This method is implemented in the R-package lmomco from Hosking. For details on this estimation technique, we refer interested readers to Hosking’s paper [18] since we use Hosking’s Fortran implementation.

### Step 4: Calculation of annual averaged wind power output generation values

The important output of algorithmic procedure are semi-empirical and estimated annual averaged wind power output generation values from from arbitrary power curves $P_{\text{Power}}$ and arbitrary wind speed probability distributions $p_{\text{Wind}}$. This calculation is based on approximations of finite integrals.

The semi-empirical averaged hourly wind power output generation value reads

(13) $$\bar{P_{Hourly,Semi-Emp.}}\approx \sum_{j=1}^{N}\frac{P_{\text{Power}}\left(v_{j}\right)}{N}$$

for all wind speed data $v_{j}\geq 0$ for all j∈{1,…,N} with physical unit $\text{kW}{\text{h}}^{-1}$. Finally, the semi-empirical averaged annual wind power output generation value is obtained by calculating

(14) $$\bar{P_{\text{Ann.,}\;\text{Semi-Emp.}}}=\frac{365\cdot 24\cdot \bar{P_{\text{Hourly,}\;\text{Semi-Emp.}}}}{1000000}$$

with physical unit $\frac{\text{GW}}{\text{year}}$. These values serve as comparative values for our estimations.

Now, we are able to approximate estimation values based on finite integrals. Let us begin with estimated hourly averaged wind power output generation values. We calculate them by

(15) $$\begin{array}{cc} \bar{P_{\text{Hourly,}\;\text{Th.}}} & =\int_{0}^{\infty }P_{\text{Power}}\left(v|\theta_{\mathrm{Power}}\right)\cdot p_{\text{Wind}}\left(v|\theta_{\text{Wind}}\right)\;\text{d}v\\ & \approx \int_{v_{\text{cut-in}}}^{v_{\text{cut-off}}}P_{\text{Power}}\left(v|\theta_{\mathrm{Power}}\right)\cdot p_{\text{Wind}}\left(v|\theta_{\text{Wind}}\right)\;\text{d}v. \end{array}$$

For example, one could use left-sided Riemannian sums

(16) $$\bar{P_{\text{Hourly,}\;\text{LRS}}}\approx \sum_{m=10\cdot v_{\text{cut-in}}}^{10\cdot v_{\text{cut-off}}}p_{\text{Wind}}\left(\frac{m}{10}|\theta_{\text{Wind}}\right)\cdot P_{\text{Power}}\left(\frac{m}{10}|\theta_{\text{Power}}\right)\cdot \frac{1}{10}$$

because wind speeds are normally measured in 0.1 steps. Other possibilities are right-sided Riemannian sums, trapezoidal approximations or Simpson’s rule. Since numerical integration is a vast field, we refer interested readers to the book by Davis and Rabinowitz [19]. This integral yields one hourly averaged wind power output generation value with physical unit $\text{kW}{\text{h}}^{-1}$. Finally, the annual averaged wind power output generation value is given by

(17) $$\bar{P_{\text{Ann.,}\;\text{Th.}}}=\frac{365\cdot 24\cdot \bar{P_{Hourly,Th}}}{1000000}$$

and the physical unit of annual averaged wind power output generation values reads $\frac{\text{GW}}{\text{year}}$.

### Step 5: Goodness-of-fit measures and uncertainty quantification

Since we want to compare different fits to curves, we often challenge the problem of comparing them. Coefficients of determination are applied to compare parametric models. Let $v_{i}\in \left[v_{\text{cut-in}},v_{\text{cut-off}}\right]$ be all measured wind speeds which are larger than the cut-in wind speed $v_{\text{cut-in}}$ and which are smaller than the cut-off wind speed $v_{\text{cut-off}}$. Denote empirical wind speed probabilities by $p_{\text{Emp.}}\left(v_{i}\right)$ and estimated wind speed probabilities of certain wind speed distribution models by $p_{\text{Wind}}\left(v_{i}\right)$. The mean of all empirical wind speed probabilities is represented by $\bar{p_{\text{Emp.}}\left(v_{i}\right)}$. The coefficient of determination reads

(18) $$R^{2}=1-\frac{\sum_{v_{i}}{\left(p_{\text{Emp.}}\left(v_{i}\right)-p_{\text{Wind}}\left(v_{i}\right)\right)}^{2}}{\sum_{v_{i}}{\left(p_{\text{Emp.}}\left(v_{i}\right)-\bar{p_{\text{Emp.}}\left(v_{i}\right)}\right)}^{2}}$$

where summations are performed over all measured wind speeds which are larger than $v_{\text{cut-in}}$ and which are smaller than $v_{\text{cut-off}}$.

We discuss error analysis on this two-parameter Weibull distributions in a more detailed manner. Our analysis relies on Taylor’s book [20]. Our starting point is (15). Assume both functions $p_{\text{Wei}}$ and $P_{\text{Power}}$ to be uncertain. Here, the wind speed probability distribution function is the two-parameter Weibull distribution. Assume that the variables $x_{1},\ldots ,x_{n}$ are measured with uncertainties $\delta x_{1},\ldots ,\delta x_{n}$ and these values are used to compute a function value $f\left(x_{1},\ldots ,x_{n}\right)$. If formula (3.48)

$$\delta f\leq \sum_{j=1}^{n}\left|\frac{\partial f}{\partial x_{j}}\right|\cdot \delta x_{j}$$

for the uncertainty δf of f from [20] is applied, the lower bound error of $p_{\text{Wei}}$ reads

$$\begin{array}{cc} p_{\text{Wei}}\left(v|A_{\text{Wei}},k_{\text{Wei}}\right)-\delta p_{\text{Wei}} & \geq p_{\text{Wei}}\left(v|A_{\text{Wei}},k_{\text{Wei}}\right)-\frac{\partial p_{\text{Wei}}\left(v|A_{\text{Wei}},k_{\text{Wei}}\right)}{\partial v}\cdot \Delta v\\ & \;\;\;\;-\frac{\partial p_{\text{Wei}}\left(v|A_{\text{Wei}},k_{\text{Wei}}\right)}{\partial A_{\text{Wei}}}\cdot \sigma_{A_{\text{Wei}}}\\ & \;\;\;\;-\frac{\partial p_{\text{Wei}}\left(v|A_{\text{Wei}},k_{\text{Wei}}\right)}{\partial k_{\text{Wei}}}\cdot \sigma_{k_{\text{Wei}}} \end{array}$$

where $\sigma_{A_{\text{Wei}}}$ and $\sigma_{k_{\text{Wei}}}$ are standard deviations of $A_{\text{Wei}}$ and $k_{\text{Wei}}$ respectively and Δv is the absolute measurement error in wind speed measurements. The lower bound error of $P_{\text{Power}}$ is given by

$$P_{\text{Power}}\left(v|\theta_{\text{Wind}}\right)-\delta P_{\text{Power}}\geq P_{\text{Power}}\left(v|\theta_{\text{Wind}}\right)-\frac{\partial P_{\text{Power}}\left(v|\theta_{\text{Wind}}\right)}{\partial v}\cdot \Delta v$$

where we neglect uncertainties in our parameter vector $\theta_{\text{Wind}}$ because we mainly apply cubic spline interpolation. Consequently, the absolute error $\Delta \bar{P_{Hourly,Th.}}$ is calculated by

(19) $$\begin{array}{cc} \Delta \bar{P_{Hourly,Th.}} & =\sum_{m=10\cdot v_{\text{cut-in}}}^{10\cdot v_{\text{cut-off}}}(\frac{1}{10}\cdot p_{\text{Wind}}\left(\frac{m}{10}|\theta_{\text{Wind}}\right)\cdot P_{\text{Power}}\left(\frac{m}{10}|\theta_{\text{Power}}\right)\\ & \;\times \{\left|\frac{\partial p_{\text{Wind}}}{\partial v}\left(\frac{m}{10}|\theta_{\text{Wind}}\right)\right|\cdot \Delta v+\left|\frac{\partial p_{\text{Wind}}}{\partial A_{\text{Wei}}}\left(\frac{m}{10}|\theta_{\text{Wind}}\right)\right|\cdot \sigma_{A_{\text{Wei}}}\\ & \;+\left|\frac{\partial p_{\text{Wind}}}{\partial k_{\text{Wei}}}\left(\frac{m}{10}|\theta_{\text{Wind}}\right)\right|\cdot \sigma_{k_{\text{Wei}}}\}+\left|\frac{\partial P_{\text{Power}}}{\partial v}\left(\frac{m}{10}|\theta_{\text{Power}}\right)\right|\cdot \Delta v\\ & \;-\left|\frac{\partial P_{\text{Power}}}{\partial v}\left(\frac{m}{10}|\theta_{\text{Power}}\right)\right|\cdot \Delta v\cdot \{\left|\frac{\partial p_{\text{Wind}}}{\partial v}\left(\frac{m}{10}|\theta_{\text{Wind}}\right)\right|\cdot \Delta v\\ & \;+\left|\frac{\partial p_{\text{Wind}}}{\partial A_{\text{Wei}}}\left(\frac{m}{10}|\theta_{\text{Wind}}\right)\right|\cdot \sigma_{A_{\text{Wei}}}+\left|\frac{\partial p_{\text{Wind}}}{\partial k_{\text{Wei}}}\left(\frac{m}{10}|\theta_{\text{Wind}}\right)\right|\cdot \sigma_{k_{\text{Wei}}}\}) \end{array}$$

as we multiply $p_{\text{Wei}}\left(v|A_{\text{Wei}},k_{\text{Wei}}\right)-\delta p_{\text{Wei}}$ and $P_{\text{Power}}\left(v|\theta_{\text{Wind}}\right)-\delta P_{\text{Power}}$ to obtain our approximate lower bound. The same argument holds for upper bounds. The first derivatives of two-parameter Weibull distributions read

$$\begin{array}{cc} \frac{\partial p_{\text{Wei}}\left(v|A_{\text{Wei}},k_{\text{Wei}}\right)}{\partial v} & =\frac{\left(k_{\text{Wei}}-1\right)\cdot k_{\text{Wei}}\cdot \text{exp}\left(-{\left(\frac{v}{A_{\text{Wei}}}\right)}^{k_{\text{Wei}}}\right)\cdot {\left(\frac{v}{A_{\text{Wei}}}\right)}^{k_{\text{Wei}}-2}}{A_{\text{Wei}}^{2}}\\ & \;\;\;\;-\frac{k_{\text{Wei}}^{2}\cdot \text{exp}\left(-{\left(\frac{v}{A_{\text{Wei}}}\right)}^{k}\right)\cdot {\left(\frac{v}{A_{\text{Wei}}}\right)}^{2\cdot k_{\text{Wei}}-2}}{A_{\text{Wei}}^{2}},\\ \frac{\partial p_{\text{Wei}}\left(v|A_{\text{Wei}},k_{\text{Wei}}\right)}{\partial A_{\text{Wei}}} & =\frac{k_{\text{Wei}}^{2}\cdot \text{exp}\left(-{\left(\frac{v}{A_{\text{Wei}}}\right)}^{k_{\text{Wei}}}\right)\cdot {\left(\frac{v}{A_{\text{Wei}}}\right)}^{2\cdot k_{\text{Wei}}}}{A_{\text{Wei}}\cdot v}\\ & \;\;\;\;-\frac{k_{\text{Wei}}^{2}\cdot \text{exp}\left(-{\left(\frac{v}{A_{\text{Wei}}}\right)}^{k_{\text{Wei}}}\right)\cdot {\left(\frac{v}{A_{\text{Wei}}}\right)}^{k_{\text{Wei}}}}{A_{\text{Wei}}\cdot v} \end{array}$$

and

$$\begin{array}{cc} \frac{\partial p_{\text{Wei}}\left(v|A_{\text{Wei}},k_{\text{Wei}}\right)}{\partial k_{\text{Wei}}} & =\frac{\text{exp}\left(-{\left(\frac{v}{A_{\text{Wei}}}\right)}^{k_{\text{Wei}}}\right)\cdot {\left(\frac{v}{A_{\text{Wei}}}\right)}^{k_{\text{Wei}}-1}}{A_{\text{Wei}}}\\ & \;+\frac{k_{\text{Wei}}\cdot \text{exp}\left(-{\left(\frac{v}{A_{\text{Wei}}}\right)}^{k_{\text{Wei}}}\right)\cdot {\left(\frac{v}{A_{\text{Wei}}}\right)}^{k_{\text{Wei}}-1}\cdot \text{ln}\left(\frac{v}{A_{\text{Wei}}}\right)}{A_{\text{Wei}}}\\ & \;-\frac{k_{\text{Wei}}\cdot \text{exp}\left(-{\left(\frac{v}{A_{\text{Wei}}}\right)}^{k_{\text{Wei}}}\right)\cdot {\left(\frac{v}{A_{\text{Wei}}}\right)}^{2\cdot k_{\text{Wei}}-1}\cdot \text{ln}\left(\frac{v}{A_{\text{Wei}}}\right)}{A_{\text{Wei}}}. \end{array}$$

Since one main goal of this article is the prediction of annual averaged wind power output generation values, absolute differences of such values are suitable comparative measures. The absolute difference between semi-empirical and estimated annual averaged wind power output generation values reads

(20) $$\Delta P_{\text{Values}}=\left|\bar{P_{\text{Ann.,}\;\text{Semi-Emp.}}}-\bar{P_{\text{Ann.,}\;\text{Th.}}}\right|.$$

## Step 6: Summary of results

All obtained data are summarized in one file. We list the important results that one might want access.

These data are saved in one file named Results_01.txt. A reduced version of collected data is saved in one file named Results_02.txt.

## Example: Bremen, Germany

We first summarize some important data regarding weather station no. 00691 located at Bremen, Germany in Table 3.

**Table 2:** Collected results

Number	Result
1	Station identification number
2	Location
3	Longitude
4	Latitude
5	Station height
6	Reading height
7	Number of data
8	Mean wind speed
9	Standard deviation of wind speed data
10	Minimum of wind speed data
11	Maximum of wind speed data
12	$k_{\text{Wei}}$
13	$A_{\text{Wei}}$
14	Semi-empirical annual averaged power output generation values
15	Estimated annual averaged power output generation values by
	Weibull distributions
16	Errors of Weibull estimates
17	Absolute differences between semi-empirical values and Weibull
	estimates
18	Estimated annual averaged power output generation values by
	Kappa distributions
19	Absolute differences between semi-empirical values and Kappa
	estimates
20	Estimated annual averaged power output generation values by
	Wakeby distributions
21	Absolute differences between semi-empirical values and Wakeby
	estimates

**Table 3:** Data for Bremen, Germany

Data	Value
Station identification number	00691
Location	Bremen, Germany
Longitude	8.80
Latitude	53.05
Station height	4.10 [m]
Reading height	10 [m]
Starting date	1926/01/01
Ending date	2018/12/31

These data is taken from a meta-data-file which accompanies the weather-station-data-file. After calculation, we obtain the following results. All these results are summarized in Table 4.

**Table 4:** Results for Bremen, Germany

Data	Results
7 from Table 2	639270
8 from Table 2	4.36 [m/s]
9 from Table 2	2.43 [m/s]
10 from Table 2	0.0 [m/s]
11 from Table 2	28.3 [ms]
12 from Table 2	1.89
13 from Table 2	4.93
14 from Table 2	3.49 [GW/year]
15 from Table 2	3.56 [GW/year]
16 from Table 2	0.23 [GW/year]
17 from Table 2	0.07 [GW/year]
18 from Table 2	3.51 [GW/year]
19 from Table 2	0.02 [GW/year]
20 from Table 2	3.53 [GW/year]
21 from Table 2	0.04 [GW/year]

**Fig. 3:**
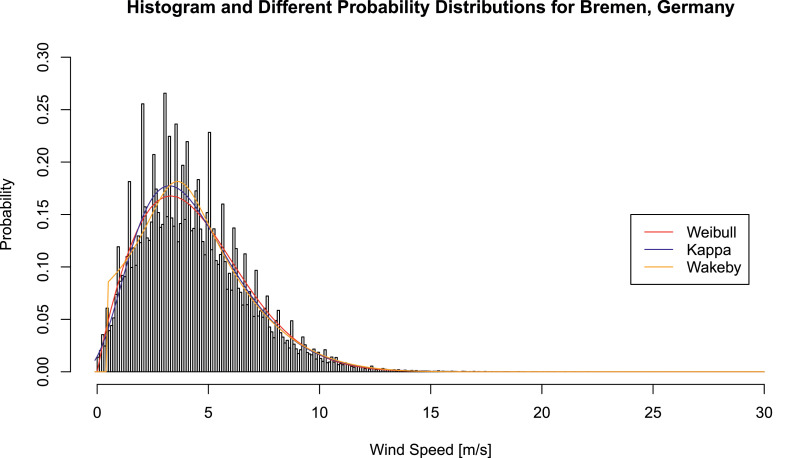
Frequency histogram and wind speed probability distributions for Bremen, Germany

The empirical histogram of wind speeds and wind speed probability distributions are portrayed in Figure 3.

**Fig. 4:**
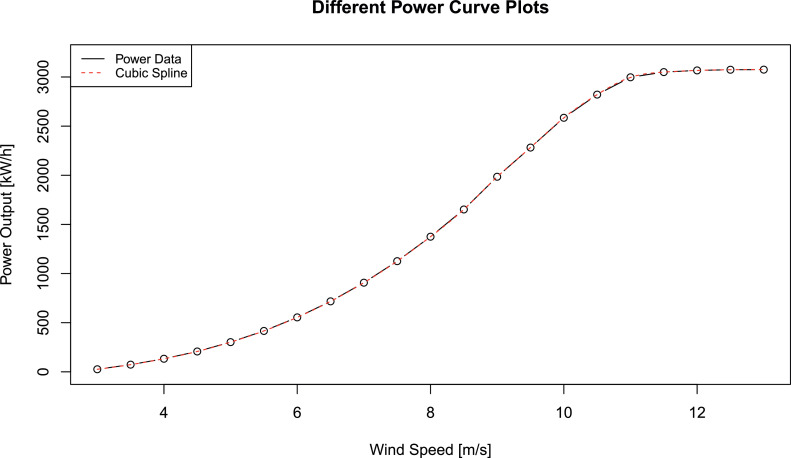
Cubic spline interpolation of power curve data

The cubic spline interpolation of power curve data is presented in Figure 4.

We conclusively report the $R^{2}$-values of all three wind speed probability distribution for this example in Table 5.

**Table 5:** $R^{2}$-values for Bremen, Germany

$R^{2}$	Value
$R_{\text{Wei}}^{2}$	0.918
$R_{\text{Kap}}^{2}$	0.922
$R_{\text{Wak}}^{2}$	0.919

## Code availability and data availability

The R [21] and GNU Octave [22] codes can be downloaded from https://github.com/bewa87/2020-Energy-AAPOGFWT. Data for the presented wind turbine from Vestas can be obtained from https://www.wind-turbine-models.com/turbines/7-vestas-v112-onshore#datasheet. Wind speed data for all German weather stations are available under [5] and wind speed data for worldwide weather stations can be accessed under [6].

## Declaration of interest

The authors declare that they have no potential conflict of interest.

## CRediT authorship contribution statement

**Benjamin Wacker:** Conceptualization, Methodology, Data curation, Validation, Visualization, Resources, Writing – original draft, Writing – review & editing. **Jan Chr. Schlüter:** Conceptualization, Resources, Writing – review & editing.
